# 5-HT2A receptor dysregulation in a schizophrenia relevant mouse model of NMDA receptor hypofunction

**DOI:** 10.1038/s41398-022-01930-0

**Published:** 2022-04-22

**Authors:** Kazuhito Nakao, Mahendra Singh, Kiran Sapkota, Andrew Fitzgerald, John J. Hablitz, Kazu Nakazawa

**Affiliations:** 1 Department of Neuroscience, Life Science Division, Southern Research, Birmingham, AL 35205 USA; 2 Department of Psychiatry and Behavioral Neurobiology, University of Alabama at Birmingham, Birmingham, AL 35294 USA; 3 Department of Neurobiology, University of Alabama at Birmingham, Birmingham, AL 35294 USA; 4 Present Address: Bristol Myers Squibb, 6-5-1 Nishi-Shinjuku, Shinjuku-ku, Tokyo 163-1328 Japan; 5 Present Address: Department of Pharmacology and Experimental Neuroscience, University of Nebraska Medical Center, Omaha, NE 68198 USA; 6 Present Address: Novartis Institutes for BioMedical Research, 250 Massachusetts Ave, Cambridge, MA 02139 USA

**Keywords:** Molecular neuroscience, Schizophrenia

## Abstract

Blockade of *N*-methyl-D-aspartate receptors (NMDAR) is known to augment cortical serotonin 2A receptors (5-HT2ARs), which is implicated in psychosis. However, the pathways from NMDAR hypofunction to 5-HT2AR up-regulation are unclear. Here we addressed in mice whether genetic deletion of the indispensable NMDAR-subunit *Grin1* principally in corticolimbic parvalbumin-positive fast-spiking interneurons, could up-regulate 5-HT2ARs leading to cortical hyper-excitability. First, in vivo local-field potential recording revealed that auditory cortex in *Grin1* mutant mice became hyper-excitable upon exposure to acoustic click-train stimuli that release 5-HT in the cortex. This excitability increase was reproduced ex vivo where it consisted of an increased frequency of action potential (AP) firing in layer 2/3 pyramidal neurons of mutant auditory cortex. Application of the 5-HT2AR agonist TCB-2 produced similar results. The effect of click-trains was reversed by the 5-HT2AR antagonist M100907 both in vivo and ex vivo. Increase in AP frequency of pyramidal neurons was also reversed by application of Gαq protein inhibitor BIM-46187 and G protein-gated inwardly-rectifying K^+^ (GIRK) channel activator ML297. In fast-spiking interneurons, 5-HT2AR activation normally promotes GABA release, contributing to decreased excitability of postsynaptic pyramidal neurons, which was missing in the mutants. Moreover, unlike the controls, the GABA_A_ receptor antagonist (+)-bicuculline had little effect on AP frequency of mutant pyramidal neurons, indicating a disinhibition state. These results suggest that the auditory-induced hyper-excitable state is conferred via GABA release deficits from *Grin1*-lacking interneurons leading to 5-HT2AR dysregulation and GIRK channel suppression in cortical pyramidal neurons, which could be involved in auditory psychosis.

## Introduction

Schizophrenia is a severe psychiatric disorder characterized by psychosis. Auditory verbal hallucination (AVH), the perception of voices in the absence of an external stimulus, is a common symptom of psychosis and is found in 60–90% of patients with schizophrenia [1, 2]. Although the neural basis of AVH is poorly understood, there is a growing interest in how cortical hyperactivity in certain brain regions may contribute to AVH experiences [3, 4]. In particular, activation of the primary auditory (A1) cortex has been reported during AVHs [5, 6]. Electrical stimulation of auditory cortex in human patients has been demonstrated to produce AVHs [7]. Moreover, recent clinical studies using a novel fMRI imaging technique with simultaneous transcranial magnetic stimulation demonstrate that impairments in GABAergic inhibition and increased cortical excitability are ubiquitous features in schizophrenia [8, 9]. Such findings support the view that impaired GABAergic inhibition and subsequent abnormal cortical activation including auditory cortex is associated with AVHs. However, the mechanisms underlying interneuron dysfunction and its contribution to cortical hyper-excitation in schizophrenia remain to be elucidated.

A mounting body of evidence suggests there is *N*-methyl-d-aspartate receptor (NMDAR) hypofunction in cortical interneurons in schizophrenia [10–17]. We have been analyzing a conditional transgenic mouse, in which the *Grin1* gene (encoding the obligatory NMDAR subunit GluN1) is disrupted in 40~50% of cortical and hippocampal interneurons, the majority of which are parvalbumin (PV)-containing fast-spiking interneurons [18]. The *Grin1* mutant mice, in which *Grin1* deletion occurs from postnatal day 7, exhibit schizophrenia-typical behaviors, such as impaired prepulse inhibition of startle reflex, deficits of spatial working memory, and exacerbation of amphetamine-induced striatal dopamine release. However, it remains to be determined whether NMDAR hypofunction in cortical GABAergic neurons confers cortical hyper-excitability.

In the search of cortical excitability, we used in vivo local field potential (LFP) recording in awake animals, and applied periodic auditory steady-state response (ASSR) stimuli, comprised of 50 repetitions of 40-Hz click-trains with inter-stimulus interval (ISI) of 20 s. As previously reported [19, 20], the *Grin1* mutant mice show diminished ASSR oscillatory power following the *N1* peak (the first negatively-evoked response). Here we found that *N1*-like spontaneous LFP activities were frequently observed during ISIs in the *Grin1* mutant mice, suggesting cortical hyperexcitability elicited by ASSR acoustic stimuli. In vivo microdialysis in awake animals also revealed that ASSR stimuli trigger 5-HT release extracellularly in the auditory cortex. Finally, the literature indicates that administration of NMDAR antagonists such as phencyclidine enhances cortical 5-HT2AR activity by assessing behavioral head-twitch responses (HTRs) and activity-dependent immediate-early gene expression levels [21–23]. These pieces of evidence led us to hypothesize that acoustic stimuli produce a 5-HT2AR-dependent hyper-excitable state in the auditory cortex of the *Grin1* mutant mice. The present study aims to test this hypothesis and further delineate the mechanism by which NMDAR hypofunction in cortical PV interneurons produces 5-HT2AR dysregulation and cortical hyper-excitability upon exposure to acoustic stimuli, which could be the basis of auditory processing dysfunction seen in the patients with schizophrenia [24].

## Materials and methods

All experimental procedures were approved by the Institutional Animal Care and Use Committee of Southern Research and University of Alabama at Birmingham. Additional information is in Supplementary Methods.

### Animals

We employed *Ppp1r2*-cre/floxed-*Grin1* KO mice (hereafter referred to as *Grin1* mutant mice), in which genetic deletion of obligatory *Grin1* subunit is introduced in 40~50% cortical and hippocampal GABAergic interneurons from ~P7 [18]. *Grin1* mutant mice were bred as a cross between *Ppp1r2*-cre line (JAX #012686) and floxed-*Grin1* line (JAX #036352) on C57BL/6J background. Homozygously-floxed-*Grin1* mice were used as control. No randomization was used to allocate animals to experimental groups.

### In vivo LFP recording

In vivo LFP recoding was conducted as previously described [19, 20]. Briefly, multi-site LFP recording was performed from A1 cortex of awake, head-restrained mice (both sexes, 10–15-week-old) in an auditory isolation chamber (background sound level, 35 dB SPL), using 6 channels of electrodes. Five hundred-ms long click-trains consisting of 80-dB white-noise pulses presented at 40 Hz (40-Hz click-train stimuli or ASSR stimuli) were applied 50 times with an inter-stimulus interval (ISI) of 20 s (~1000 s). For analysis of spontaneous LFP activities, data were used when the first negatively-evoked (N1) responses with an amplitude greater than 0.1 mV (~4 times the standard deviation) were observed in more than two channels in an animal. Spontaneous LFP activities were counted during ISIs (20-s each repeated 49 times, per animal) when *N1*-like negative discharges were simultaneously elicited in more than two channels with varying amplitudes of at least over 0.05 mV (~2 times the standard deviation). The selective 5-HT2AR antagonist M100907 (0.3 mg/kg, Sigma) [25, 26] or the selective Girk1/2-containing G protein–coupled inwardly rectifying K^+^ channels (GIRK) channel activator ML297 (30 mg/kg, Tocris) [27, 28] was intraperitoneally (IP) injected 30 min before in vivo LFP recordings to examine their effect on LFP activities. The sample size (*n* = 5) was determined by the power analysis. Male and female data were mixed as no differences were observed.

#### In vivo brain microdialysis

In vivo brain microdialysis was conducted as previously described [29]. Briefly, a microdialysis CMA7 probe was inserted into right auditory cortex through a guide cannula. The mouse was then placed into a test chamber. Following a 2-h equilibration period, samples were collected every 20 min at 1 µl/min perfusion rate into a microcentrifuge tube containing antioxidants. The samples were collected before and after application of ASSR stimuli and local infusion of saline or glutamatergic blockers [100 µM NBQX (Alomone Labs) and 50 µM 3-((R)-2-Carboxypiperazin-4-yl)-propyl-1-phosphonic acid (R-CPP), Sigma]. Samples (*n* = 4 or 5 for each genotype) were analyzed by HPLC.

### Head twitch responses

After administration of DOI (2,5-Dimethoxy-4-iodoamphetamine; 0.5 mg/kg, i.p., Sigma) [30–32] or 5-Hydroxytryptophan (precursor of serotonin, 100 mg/kg, IP, Sigma) [32], the mice were placed in a 1000 ml glass beaker. Their behavior was recorded for 30 min at 240 fps using high-speed digital video camera (Olympus TG-5 camera, Japan) [31]. The MPEG-4 files were transferred to PC, and then the number of HTR were counted at 60 fps or 90 fps by Power DVD 20 (Cyber Link, US). Data were scored by the experimenters, who were blind to the genotypes.

### Ex vivo whole-cell patch-clamp recording

#### Brain slice preparation

Before slice preparation, some animals individually received 40-Hz click-train stimuli in a sound-proof chamber and compared with littermates receiving no click-train stimuli. Within 10–15 min after the cessation of acoustic stimuli, coronal brain slices containing auditory cortex were prepared. The sample size was 5–13 cells from 3–4 mice (both sexes, 4–6 week-old) as described [20].

#### Patch clamp electrophysiology

Whole-cell voltage/current clamp recordings were obtained from layer 2/3 (L2/3) pyramidal neurons as described previously [20, 33]. The recordings from L2/3 PV interneurons were obtained in tdTomato/G42-GFP double-positive neurons under *Ppp1r2*-cre/floxed-*Grin1*(f/f) or *Ppp1r2*-cre mouse strain background, as described in Supplementary Methods. Neuronal responses to a series of current injections ranging from −200 pA to 400 pA with step of 25 pA were recorded in current clamp mode. To record spontaneous inhibitory postsynaptic current (sIPSC), whole-cell voltage clamp recordings were done on layer 2/3 pyramidal neurons holding the membrane potential at −70 mV. IPSCs were isolated by adding 50 µM D-AP5 and 20 µM CNQX to the recording artificial CSF. IPSCs were detected and analyzed using custom written Igor procedures by setting event detection threshold at 5 pA. To achieve pharmacological manipulation, the following chemicals were bath-applied for minimum of 10 min during the electrophysiological recording; 5-HT2AR agonist TCB-2 (20 µM, Tocris) [34, 35], 5-HT2AR antagonist M100907 (2 µM, Sigma) [36–38], 5-HT2A/2CR antagonist Ketanserin (0.2 µM, Tocris) [39], 5-HT 2 C receptor agonist MK212 (2 µM, Tocris) [40–42], GIRK1/2 channel activator ML297 (10 µM, Tocris) [43, 44], Gαq-selective G protein inhibitor BIM-46187 (50 µM, Calbiochem) [45, 46], GABA_A_ receptor antagonist (+)-bicuculline (20 µM, Alomone Labs) [47]. Each experiment was repeated and replicated at least three times. The variance between the groups statistically compared was similar.

### Statistical analyses

Statistical analyses were conducted using JASP (version 0.14.1) (University of Amsterdam open-source data analysis software) and Prism (GraphPad Software, Inc.). Student’s *t*-test (two-sided), paired *t*-test and factorial analysis of variance (ANOVA) were employed where appropriate. When main effects or interaction effects were significant, Tukey-Kramer post hoc analysis was conducted to determine which groups differ significantly from other groups. Data are presented as mean ± s.e.m. Significance was considered at *p* < 0.05.

## Results

### Spontaneous LFP activities in mutant auditory cortex upon acoustic stimuli

**Fig. 1:**
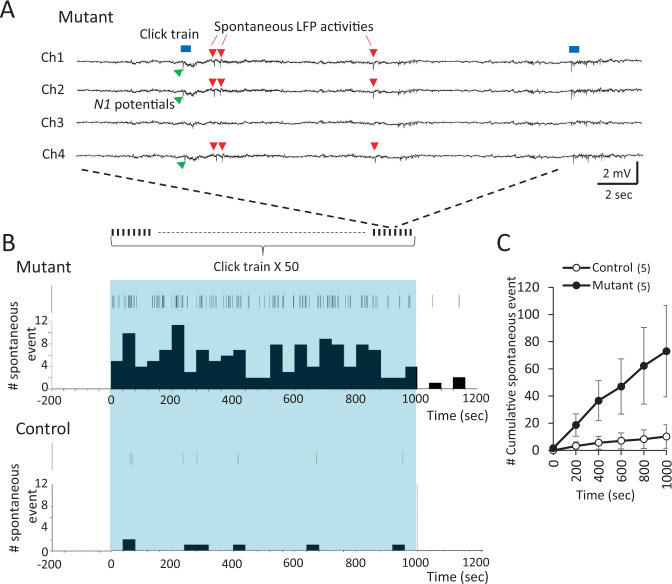
Spontaneous local field potentials (LFPs) induced by auditory stimulation. **A** Representative examples of in vivo LFPs from four electrode channels in the auditory cortex of awake head-restrained *Grin1* mutant mice before and after 40-Hz click-train stimulations. Each 40-Hz click-train (blue square, 500-ms long each) induced auditory-evoked *N1* potentials (green arrowheads) followed by ASSRs. Notably, spontaneous LFP activities (red arrowheads) were observed across channels during ISIs of 20 s mostly in the mutant mice. **B** Representative raster plots and the histograms of the spontaneous LFP activities in *Grin1* mutant mice (top) and in the floxed-control mice (bottom) during ISIs throughout the recording periods. Blue area is the period of periodic click-train application (50x) every 20 s. Note that no spontaneous spike activity was detected in the mutants (and controls) prior to the first click-train application. **C** Cumulative number of the spontaneous LFP activities (events) across ISIs in *Grin1* mutant mice and control mice (13–15-week-old, both sexes). Spontaneous activities appeared much more frequently in the mutants compared to controls (Mutants vs Controls, *F*(1, 392) = 11.03, *p* = 0.014, Repeated measures ANOVA, Tukey-Kramer post hoc test). Data are mean ± sem. Animal number in parenthesis.

To address whether *Grin1* deletion in cortical GABAergic interneurons produces a hyper-excitable state in the auditory cortex, in vivo LFP recording was performed from A1 cortex of awake *Grin1* mutant mice. When periodic 40-Hz click-train stimuli were presented as ASSR acoustic stimuli, the evoked LFP power amplitudes appeared at 35–44 Hz, which were phase-locked to the click trains in the floxed-*Grin1* control mice. We reported previously that the ASSR power amplitudes and their phase-locking were diminished in the *Grin1* mutant mice [19, 20]. Here we further found in the similar recordings that robust *N1*-like spontaneous LFP activities appeared during the 20-s ISIs between ASSR stimuli (Fig. 1A, B). These LFP activities are not epileptiform discharges, because their spike amplitudes were different across the channels, presumably due to the distance between each channel electrode and the site of spike generation. Conversely, almost no such spontaneous LFPs were detected in the pre-stimulus period prior to the first acoustic stimulus in the mutant mice, or in any recording periods in the floxed-control mice. The cumulative numbers of spontaneous LFP events were higher in the mutant mice (Fig. 1C). Spontaneous LFP events were occasionally detected for at least 30–60 min after the cessation of acoustic stimuli. These results suggested that *Grin1* deletion in GABAergic interneurons in the auditory cortex transiently induces a hyper-excitable state upon acoustic stimulation.

**Fig. 2:**
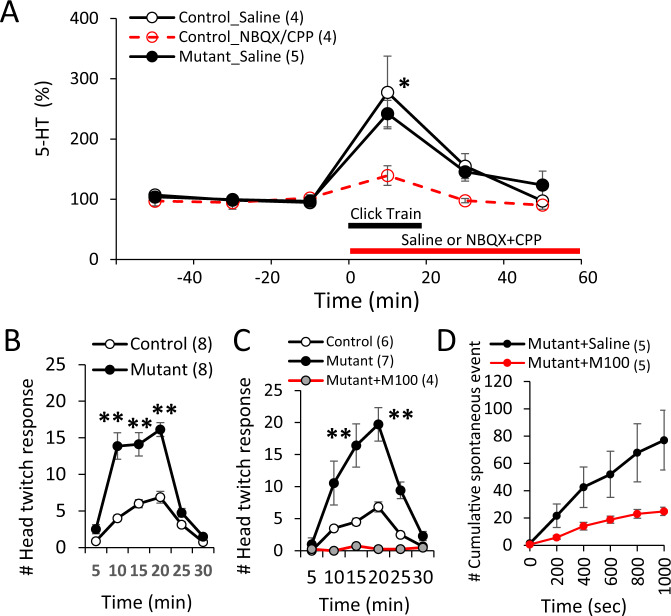
Spontaneous LFP activities mediated by 5-HT2AR activation. **A** In vivo awake microdialysis revealed that both control mice (*n* = 4) and mutant mice (*n* = 5, 10–12 week-old, both sexes) showed an increase in extracellular 5-HT up to 250–280% levels in the auditory cortex during periodic 40-Hz click-trains, which was blocked by reverse dialysis of 100 µM NBQX and 50 µM CPP (*n* = 4) (Control_Saline vs Control_NBQX/CPP, *p < 0.05, Student’s *t*-test). **B**, **C** *Grin1* mutant mice (10–12-week-old, both sexes) exhibited much higher numbers of HTRs to 5-hydroxytryptophan (5-HTP; 100 mg/kg, *i.p*.) or **C** DOI (0.5 mg/kg, *i.p*.) compared to the same-age control mice (Tukey-Kramer *post hoc* test, **p < 0.01). Note that DOI-mediated HTRs in the mutants (*n* = 4) were blocked by simultaneous treatment with 5-HT2AR antagonist M100907 (0.3 mg/kg, i.p.). HTRs were counted from zero min to 30 min after 5-HTP or DOI administration. Neither genotypes showed HTRs before the treatments. **D** In vivo awake LFP recordings exhibited that spontaneous LFP activities during ISIs in the mutant auditory cortex are suppressed by M100907 (0.3 mg/kg, i.p.) pretreated 30-min prior to the first click-train compared to the *Grin1* mutant mice with saline treatment (*n* = 5 each, *F*(1, 392) = 5.52, *p* = 0.039, Repeated measures ANOVA, Tukey-Kramer post hoc test). Data are mean ± sem. Animal number in parenthesis (both sexes).

To explore potential mediators inducing stimulus-induced excitability increases, we measured serotonin (5-hydroxytryptamine, 5-HT) and dopamine levels during acoustic stimulation by in vivo microdialysis, each of which is known to regulate cortical excitability [48, 49]. Acoustic click-train stimulation increased auditory cortex 5-HT levels in both controls and mutants (Fig. 2A), but not in the medial PFC (Fig. S1A). This 5-HT release in the auditory cortex was prevented by reverse dialysis of glutamate blocker cocktail (AMPA receptor blocker NBQX and NMDAR blocker CPP), suggesting that 5-HT release is regulated at the serotonergic fiber terminals in a glutamatergic activity-dependent manner. No change was detected in the dopamine levels in both genotypes (controls, 103% change; mutants, 102% change).

We next measured HTRs, a paroxysmal side-to-side rapid head rotation mediated by cortical 5-HT2AR activation [50]. We found that HTRs were augmented in *Grin1* mutant mice, following *i.p*. administration of 5-Hydroxytryptophan (a precursor of 5-HT) and 5-HT2A/2 C receptor agonist DOI (Fig. 2B, C). DOI-induced HTRs in mutants were blocked by simultaneous treatment with 5-HT2AR antagonist M100907, suggesting up-regulation of cortical 5-HT2ARs in the mutant cortex. To further explore whether cortical hyper-excitability is 5-HT2AR-mediated, we examined the effect of M100907 on spontaneous LFP activities (Fig. 2D). Intraperitoneal injection of M100907 30 min before application of acoustic stimuli greatly suppressed the spontaneous LFP activities, suggesting cortical hyper-excitable state is mediated by 5-HT release and subsequent 5-HT2AR activation in the mutant auditory cortex.

### 5-HT2AR activation increases excitability in mutant cortical pyramidal neurons

**Fig. 3:**
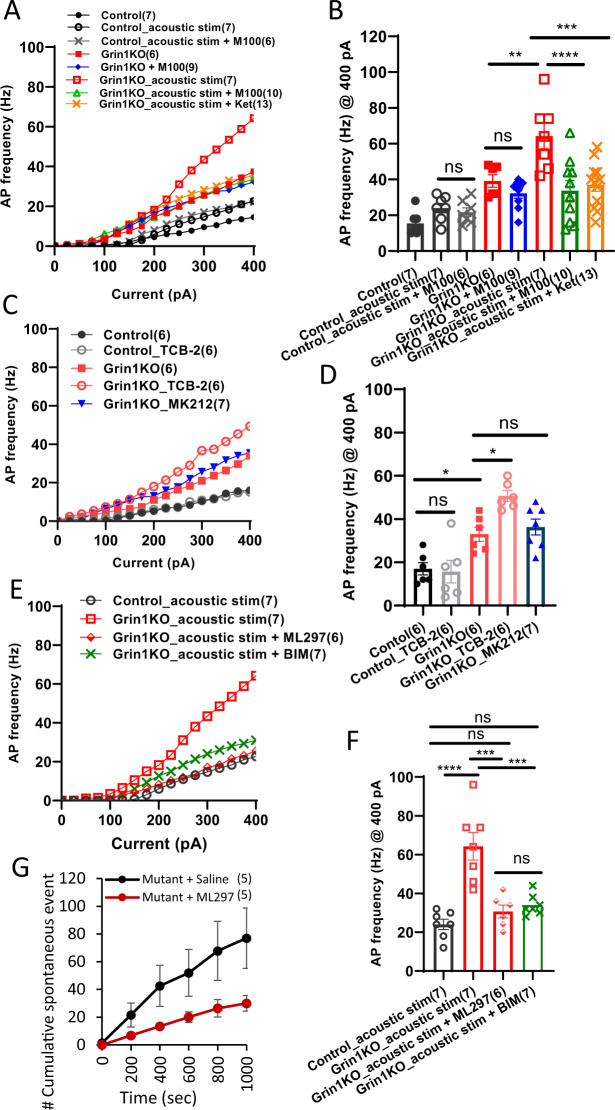
Effect of acoustic stimulation, 5-HT2AR activation, Gαq-protein inhibition and GIRK channel activation on AP firing in auditory cortex pyramidal neurons. **A** Mean AP frequency of pyramidal neurons as function of current injection (zero to 400 pA, step current 25 pA). Ex vivo recordings were made from the mice with or without drug/acoustic stimulation in vivo. **B** Summary plot of AP frequency in response to 400 pA-current injection. *Grin1* mutants exhibited higher AP frequency than controls [15.4 ± 2.4 Hz for control (*n* = 7 from 4 mice) vs 39.2 ± 3.5 Hz for mutant (*n* = 6 from 3 mice), *p = 0.017] which was further increased by acoustic stimulation [39.2 ± 3.5 Hz for mutant (*n* = 6 from 3 mice) vs 64.3 ± 7.1 Hz for mutant with acoustic stimuli, **p = 0.0096]. Acoustic stimulation had no effect on AP frequency in control mice [15.4 ± 2.4 Hz for control (*n* = 7 from 4 mice) vs 24.0 ± 2.7 Hz for control with acoustic stimuli, p = 0.86]. Increase in AP frequency in the mutants was attenuated by 5-HT2AR antagonist M100907 (M100; 2 µM) [64.3 ± 7.1 Hz for mutant with acoustic stimuli (*n* = 7 from 3 mice) vs 33.8 ± 5.6 Hz for mutant with acoustic stimuli_M100 (*n* = 10 from 3 mice), ****p < 0.0001] and by 5-HT2A/2CR antagonist Ketanserin (Ket; 0.2 µM) [64.3 ± 7.1 Hz for mutant with acoustic stimuli (*n* = 6 from 3 mice) vs 37.0 ± 3.6 Hz for mutant with acoustic stimuli_Ket (*n* = 13 from 3 mice), ***p = 0.0004]. **C** Effect of 5-HT2AR agonist TCB-2 on mean AP frequency of pyramidal neurons as function of current injection. **D** Summary plot of AP frequency in response to 400 pA-current injection. Bath-application of TCB-2 increased AP frequency in mutant mice [33.0 ± 3.3 Hz for mutant (*n* = 6 from 3 mice) vs 50.6 ± 2.5 Hz for mutant with TCB-2 (*n* = 6 from 3 mice), *p = 0.014] but not in floxed-control mice [17.0 ± 2.9 Hz for control (*n* = 6 from 3 mice) vs 15.6 ± 5.2 Hz for control with TCB-2 (*n* = 6 from 3 mice), *p* = 0.80]. MK212, a selective agonist of 5-HT2CR, had no effect on AP frequency of the mutant pyramidal neurons [33.0 ± 3.3 Hz for mutant (*n* = 6 from 3 mice) vs 36.3 ± 3.7 Hz for mutant with MK212 (*n* = 7 from 3 mice), *p* = 0.77]. *p < 0.05, One-way ANOVA. **E** AP firing frequency as function of current injection after acoustic stimulation in the presence or absence of GIRK1/2 channel activator ML297 (10 µM) and Gαq-protein inhibitor BIM-46187 (50 µM). **F** Summary plot of AP frequency in response to 400-pA current injection. Acoustic stimulation-induced increase in AP frequency was attenuated by ML297 and BIM-46187 (24.0 ± 2.6 Hz for control (*n* = 7 from 3 mice), -vs 64.29 ± 7.12 Hz for mutant (*n* = 7 from 3 mice), ***p = 0.0001; - vs 30.7 ± 3.3 Hz for mutant with ML297 (*n* = 6 from 3 mice), *p* = 0.50; -vs 34.0 ± 2.0 Hz for mutant with BIM-46187 (*n* = 7 from 3 mice), *p* = 0.29. Bar diagram represents mean ± sem in **B**, **D**, and **F**. **G** In vivo awake LFP recording revealed that pretreatment with ML297 (30 mg/kg, i.p.) 30-min prior to the onset of acoustic stimuli, reduced the cumulative numbers of spontaneous LFP activities in *Grin1* mutant mice (Mutant + Saline vs Mutant + ML297, *n* = 5 each, *F*(1, 392) = 3.67, *p* = 0.041, Repeated measures ANOVA, Tukey-Kramer post hoc test). Data are mean ± sem.

To delineate the cellular mechanism underlying auditory-triggered cortical hyper-excitability, ex vivo recordings were made from the mice receiving ASSR acoustic stimuli in vivo in the sound-proof chamber, and compared to the stimulus-free animals. Whole-cell patch-clamp recordings were obtained from auditory L2/3 pyramidal neurons presumably involved spontaneous LFP generation [51]. Intrinsic excitability was examined by injecting -200 pA to 400 pA currents with step size of 25 pA. Naïve *Grin1* mutant mice showed an increased number of APs compared to the floxed-controls (Fig. 3A, B). This excitability increase was unaffected by M100907, suggesting that excitability of the mutant pyramidal neurons was constitutively (i.e., agonist-independently) enhanced. We further found in the mutants that this AP frequency increase was greatly exacerbated by acoustic stimulation. This increase in intrinsic excitability was completely reversed by M100907 and by 5-HT type-2A and -2C receptor (5-HT2A/CR) antagonist Ketanserin, suggesting 5-HT2AR-mediated excitability increase. In contrast, AP frequency in floxed-control mice was unaltered by the acoustic stimulation or the stimuli with concomitant application of M100907. There were no major changes in other parameters of membrane excitability of L2/3 pyramidal neurons before and after acoustic stimuli (Table S1).

We next examined whether the effect of acoustic stimulation can be induced by bath-application of 5-HT2AR agonist TCB-2. Similar to the change in AP frequency of mutant pyramidal neurons following acoustic stimuli, TCB-2 increased AP frequency in mutant mice, but not in floxed-control mice (Fig. 3C, D). In contrast, bath-application of 5-HT type-2C receptor (5-HT2CR) agonist MK212, another cortical excitatory 5-HTR modulating HTRs [52], did not produce any change in AP frequency of the mutant pyramidal neurons. These results suggest that the hyper-excitable state in mutant pyramidal neuron is mediated, at least in part, by selective activation of 5-HT2ARs.

### Effect of BIM-46187 and ML297 on pyramidal neuron excitability

Activation of 5-HT2ARs in cortical PV-positive interneurons is coupled to the Gq subfamily of Gα proteins which suppress GIRK channels, leading to increased excitability [53]. Although it cannot form functional homomers [54], GIRK1 is an integral subunit of the neuronal GIRK channels [55]. We examined whether the acoustic stimuli-triggered excitability increase in L2/3 pyramidal neurons is also attributable to suppression of GIRK channels in mutant pyramidal neurons. The GIRK1/2 channel activator ML297 was bath-applied to the brain slices prepared from animals which had received acoustic stimulation in vivo. AP frequency in pyramidal neurons from mutant mice was reduced to the level comparable to floxed-controls by ML297 (Fig. 3E, F). The Gq subfamily-selective Gα-protein inhibitor BIM-46187 also suppressed the excitability, suggesting that acoustic stimuli-triggered activation of 5-HT2ARs in the pyramidal neurons inhibits GIRK channels via Gq-subtype Gα proteins. We also found that pretreatment of ML297 greatly decreases the frequency of spontaneous LFP activities during the ISIs under the ASSR protocol, suggesting that suppression of GIRK channels by 5-HT2AR activation also occurs in vivo (Fig. 3G).

### Lack of 5-HT2AR-mediated facilitation of GABA release in mutant mice

**Fig. 4:**
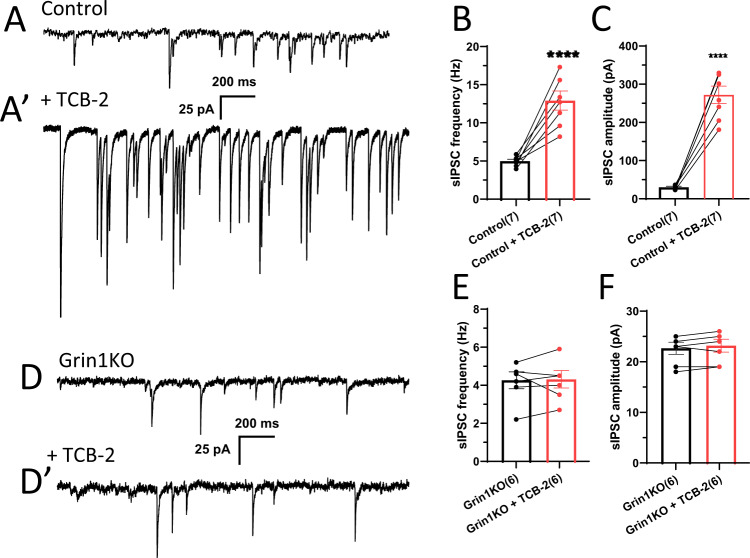
Effect of 5-HT2AR agonist TCB-2 on spontaneous IPSCs in L2/3 pyramidal neurons. **A**, **A′** Representative traces of sIPSCs before and after application of 5-HT2AR agonist TCB-2 (20 µM). **B**, **C** Robust increase in frequency [4.9 ± 0.2 Hz before TCB-2, vs 12.6 ± 1.2 Hz after (*n* = 6 from 3 mice), ****p < 0.0001] and amplitude [28.3 ± 2.2 pA before TCB-2 vs 26.3 ± 22.1 pA after (*n* = 6 from 3 mice), ****p < 0.0001] of sIPSCs from pyramidal neurons in the floxed-control mice after TCB-2 bath-application. **D**, **D′** Representative traces of sIPSCs made from *Grin1* mutant mice. No alteration in frequency [**E**; 4.1 ± 0.4 Hz before TCB-2, vs 4.2 ± 0.4 Hz after (*n* = 5 from 3 mice), *p* = 0.93] and amplitude [**F**; 22.0 ± 1.2 pA before TCB-2, vs 22.5 ± 1.2 pA after (*n* = 5 from 3 mice), *p* = 0.77] of sIPSCs in *Grin1* mutant slices by TCB-2 treatment. Bar diagrams represent mean ± sem, ***p < 0.001, p**** < 0.0001, Student’s *t* test.

5-HT release by acoustic stimulation may augment presynaptic inhibition by facilitating GABA release from PV interneurons, as previously reported [56]. Indeed, spontaneous IPSC (sIPSC) recording in L2/3 pyramidal neurons revealed that bath-application of TCB-2 increased both sIPSC frequencies and amplitudes in the controls (Fig. 4A–C). Notably, this TCB-2 induced increase of sIPSCs was absent in the mutant mice (Fig. 4D–F). Before TCB-2 application, frequency and amplitude of sIPSCs were similar to the controls, which was consistent with a recent report in mPFC pyramidal neurons [57]. These findings suggest down-regulation of presynaptic 5-HT2ARs in the mutant PV interneurons. Fluorescence in situ hybridization (FISH) showed that levels of 5-HT2AR mRNA in the somata of *Grin1*-deleted PV interneurons were reduced by ~25% compared to controls (*p* < 0.04, Student’s *t* test) (Fig. S2A). Immunoreactivity against 5-HT2AR was also decreased by ~15% in the mutant PV interneurons (*p* < 0.01) (Fig. S2B).

### Impaired presynaptic inhibition contributes to excitability increase of mutant pyramidal neurons

**Fig. 5:**
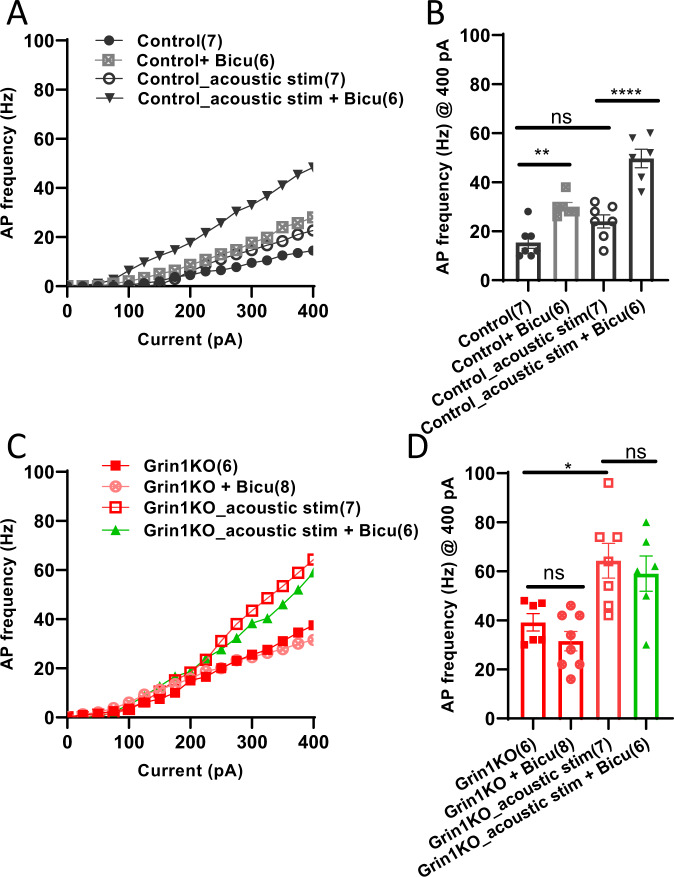
Effect of GABA receptor blockade on pyramidal neuron AP firing frequency. **A**, **B** Application of (+)-bicuculline (Bicu, 20 µM) increased AP frequency in the floxed-control mice with no acoustic stimulation [15.4 ± 2.5 Hz for control (*n* = 7 from 4 mice) vs 30.0 ± 1.7 Hz for control with Bicu (*n* = 6 from 3 mice), **p = 0.0032] and following acoustic stimulation [24.0 ± 2.7 Hz for control (*n* = 7 from 3 mice) vs 49.7 ± 3.8 Hz for control with Bicu (*n* = 6 from 3 mice), ****p < 0.0001]. **C**, **D** Application of bicuculline failed to increase the AP frequency in the mutant mice not only following acoustic stimulation [64.3 ± 7.1 Hz for Grin1KO (*n* = 7 from 3 mice) vs 59.0 ± 7.2 Hz for Grin1KO with Bicu (*n* = 6 from 3 mice), *p* = 0.91], but also without acoustic stimulation [39.2 ± 3.5 Hz for Grin1KO (*n* = 6 from 3 mice) vs 31.5 ± 3.9 Hz for Grin1KO with Bicu (*n* = 8 from 4 mice), *p* = 0.76]. Bar diagram represents mean ± sem. *p < 0.05, **p < 0.01, ****p < 0.0001. One-way ANOVA.

Lastly, we explored whether lack of 5-HT-triggered facilitation of GABA release from PV interneurons is implicated in disinhibition of mutant L2/3 pyramidal neurons. Bath-application of a GABA_A_ receptor antagonist (+)-bicuculline increased the excitability of L2/3 pyramidal neurons in the naïve control mice (Fig. 5A, B). This increase was greatly enhanced following acoustic stimulation, suggesting a significant contribution of 5-HT-mediated facilitation of GABA release to the presynaptic inhibition in controls. Thus, there are two levels of presynaptic inhibitions; constant inhibition at the basal level and on demand inhibition due to *Grin1*- and 5HT2AR-dependent GABA release. If the former inhibition was intact and the latter inhibition was disturbed in the *Grin1* mutants, it could potentially argued that the lack of -5HT2AR-mediated GABA release is implicated in the disinhibition. However, we found that treatment with (+)-bicuculline had no effect on AP frequency of the mutant pyramidal neurons, not only following acoustic stimulation, but in the naïve mutants (with no stimulation) (Fig. 5C, D), indicating presynaptic inhibition is defective regardless of 5-HT increase. The results rather suggest a deficit in GABA release machinery in *Grin1*-deleted PV neurons, which may explain the impaired presynaptic inhibition of mutant pyramidal neurons.

## Discussion

The present study delineates possible neural mechanisms of acoustic stimulus-induced cortical hyper-excitability in a mouse model of NMDAR hypofunction in cortical PV interneurons. The results suggested that 5-HT is transiently released in the auditory cortex by acoustic stimuli, which activates excitatory 5-HT2ARs located in cortical pyramidal neurons. In the controls, 5-HT also activated 5-HT2ARs in PV interneurons, releasing GABA and contributing to presynaptic inhibition. Such strong presynaptic inhibition prevented membrane depolarization by excitatory 5-HT2AR activation in the pyramidal neurons. Conversely, *Grin1* deletion in PV-positive interneurons disrupted 5-HT-triggered facilitation of GABA release. However, the pyramidal neurons were fully disinhibited even without the acoustic stimuli, implying a more profound deficit in presynaptic GABA release per se under naïve condition. This impaired presynaptic inhibition may ultimately dysregulate the postsynaptic 5-HT2ARs in mutant pyramidal neurons. Upon acoustic stimuli-releasing 5-HT, activation of the dysregulated 5-HT2ARs produced a hyper-excitable state in cortical pyramidal neurons by suppressing GIRK channels via inhibitory Gαq protein, leading to emergence of spontaneous LFP activities (see Fig. S3 for schematic model).

### Acoustic stimuli increase extracellular 5-HT level in auditory cortex

In vivo microdialysis in awake animals revealed that extracellular 5-HT concentration is increased in the auditory cortex upon acoustic stimulation (Fig. 2A). This finding is consistent with previous reports demonstrating that acoustic stimuli increase the activity of tryptophan hydroxylase (5-HT synthesizing enzyme) and 5-HT turnover in the medial raphe nuclei [58–60]. Considering a critical role of 5-HT in the biological effects of stress resilience or coping [61, 62], it is tempting to speculate that acoustic stimuli acts as a sound stressor for releasing 5-HT. Numerous stressors, such as emotional (restraint, loud noise, skin pinching) and metabolic (hypoglycemia) stimuli, are shown to increase extracellular 5-HT at the level of serotonergic cell bodies or nerve terminals [63]. Notably, 5-HT was not increased in mPFC by the acoustic stimuli (Fig. S1A), so that acoustic stimuli had no effect on the AP frequency of mPFC L2/3 pyramidal neurons (Fig. S1C, D). Conversely, immobilization stress for 60 min triggers 5-HT increase in mPFC but not in the auditory cortex (Fig. S1B). These results raise a possibility that cortical areas where 5-HT is released depend on the types of stress-inducing stimuli. We further found the local infusion of glutamate blockers into the auditory cortex mostly suppressed stimuli-induced 5-HT release locally (Fig. 2A). In fact, previous studies have questioned a direct links between 5-HT release and the cell firings in the raphe nuclei [63–65]. 5-HT release may be regulated by glutamatergic inputs terminating at the serotonergic fiber terminals.

### Down-regulation of 5-HT2ARs in *Grin1*-deleted PV interneurons

The major finding of our study is that *Grin1* deletion in immature PV interneurons produces a hyper-excitable state upon acoustic stimulation in the auditory cortex pyramidal neurons. This cortical hyper-excitability originated from impaired GABAergic inhibition by *Grin1*-deleted PV interneurons. 5-HT is known to facilitate GABA release by activating 5-HT2ARs of cortical PV-positive interneurons [56, 66–68]. We found that *Grin1* deletion in immature PV interneurons abolishes 5-HT2AR-mediated facilitation of GABA release from PV interneurons, as assessed by sIPSCs (Fig. 4). This lack of 5-HT2AR agonist response could be due to the down-regulation of 5-HT2ARs in PV interneurons (Fig. S2). However, we also found that increased excitability of mutant pyramidal cells is insensitive to (+)-bicuculline even without acoustic stimulation. Therefore, it is unclear to what extent 5-HT-dependent GABA release deficit can contribute to dysregulation of postsynaptic 5-HT2ARs. Regardless, mutant PV interneurons were unable to hyperpolarize postsynaptic pyramidal neurons in response to acoustic stimuli (Fig. 5C, D), which may contribute to the emergence of spontaneous LFP activities following acoustic stimulation. Cellular mechanism of GABA release deficit in *Grin1*-deleted PV interneurons remains to be determined.

### Up-regulation of 5-HT2ARs in cortical pyramidal neurons

*Grin1*-deletion in immature PV interneurons also altered 5-HT2AR function in postsynaptic pyramidal neurons. Normally, excitability of cortical pyramidal neurons is regulated by a balance between excitatory 5-HT2ARs and inhibitory 5-HT1ARs [69], which are co-expressed in a large fraction of cortical pyramidal neurons including L2/3 cells [48], and the hyperpolarizing effect of 5-HT is dominant on membrane potential in adult animals [69, 70]. In fact, application of acoustic stimuli or TCB-2 did not alter the AP frequency in control pyramidal neurons (Fig. 3). Conversely, ex vivo patch-clamp recording in *Grin1* mutant mice revealed a robust increase in AP frequency of L2/3 pyramidal neurons following acoustic stimulation. This excitability increase in mutants was also observed by TCB-2 application alone. Furthermore, pretreatment with 5-HT2AR antagonist M100907 greatly suppressed the AP frequency ex vivo (Fig. 3A) and spontaneous LFP activities in vivo (Fig. 2D). Enhancement of HTRs by DOI (5-HT2A/2CR agonist) treatment in the mutant mice was abolished by M100907 (Fig. 2). All the data suggested up-regulation of 5-HT2AR function in the mutant cortical pyramidal neurons. Although the evidence for 5-HT2ARs coupled to GIRK channels in pyramidal neurons is normally scarce [71], we found that the increased excitability in the pyramidal neurons is, at least partly, attributed to suppression of GIRK channels via Gαq proteins. Selective GIRK channel activator ML297 and Gαq inhibitor BIM-46187 both reduced the excitability to the levels of pyramidal neurons in controls (Fig. 4A). ML297 also largely suppressed the emergence of spontaneous LFP activities induced by acoustic stimuli in vivo (Fig. 4E). Suppressing downstream activity of 5-HT2ARs by these compounds may potentially be an effective strategy to reduce psychosis.

Notably, AP frequency of pyramidal neurons with no TCB-2 or acoustic stimuli (*i.e*., baseline level) was already higher in the mutants compared to the control mice. This excitability increase in naïve mutant animals, presumably caused by long-lasting disinhibition, appears to be independent of 5-HT binding to 5-HT2ARs, because the competitive antagonist M100907 reversed only to the level of mutant AP frequency, but not to the level of controls. Mutant 5-HT2ARs could potentially be, to some degree, constitutively active in the absence of agonist in the pyramidal neurons, as previously suggested [72], leading to the increased excitability by constant suppression of GIRK channels. In fact, treatment of GIRK1/2 channel activator ML297 decreased the AP frequency down to the level of control cells (Fig. 4A). Further research is needed to determine the mechanisms by which 5-HT2AR in mutant pyramidal neurons is up-regulated without agonist stimulation.

### Abnormalities of both serotonin and dopamine neuromodulator systems

Three different pharmacological mechanisms have been proposed for the basis of clinical psychosis: activation of striatal dopamine D2R with psychostimulants, activation of cortical 5-HT2AR with psychedelics, and blockade of NMDAR with dissociative anesthetics [73]. It has been long debated how these distinct mechanisms interact with each other to elicit psychosis. Animal studies showed that administration of non-competitive NMDAR antagonists augments striatal dopamine release [74–76] and potentiate cortical 5-HT2AR function [21–23], suggesting that systemic blockade of NMDARs promotes both striatal dopamine hyperactivity and cortical 5-HT2AR activation. Furthermore, we recently showed an exacerbation of amphetamine-induced striatal dopamine release in the *Grin1* mutant mice [29]. The present study demonstrated that the same *Grin1* mutant mice augment cortical 5-HT2AR function in response to the 5-HT releasing stimuli. These findings suggest a crucial role of NMDAR hypofunction played by PV interneurons in the auditory psychosis.

## Conclusion

The results of present study demonstrate that NMDAR hypofunction in cortical PV-positive interneurons confers two contrasting altered 5-HT2AR responses to 5-HT, lack of response in *Grin1*-deleted PV interneuron terminals and greater intrinsic excitability in the pyramidal neurons. Since up-regulation of 5-HT2ARs in pyramidal neurons has been proposed as a common neural mechanism that could predispose to psychosis [77, 78], the *Grin1* mutant mouse can be a suitable model for research into the pathophysiology of psychosis in schizophrenia and other types of psychotic disorders.

## Supplementary information

Nakao K et al._Supplementary Information
