# Supplementary material for: 5-HT2A receptor dysregulation in a schizophrenia relevant mouse model of NMDA receptor hypofunction
Source: Transl Psychiatry. 2022 Apr 22;12:168. doi: 10.1038/s41398-022-01930-0 (PMC9033804; doi:10.1038/s41398-022-01930-0)
Supplement: Supplementary file 1 — Nakao K et al._Supplementary Information [file 41398_2022_1930_MOESM1_ESM.pdf]

5-HT2A Receptor Dysregulation in a Schizophrenia Relevant Mouse Model of NMDA Receptor Hypofunction

Kazuhito Nakao, Mahendra Singh, Kiran Sapkota, Andrew Fitzgerald, John J Hablitz and Kazu Nakazawa

Supplementary Methods

Labeling of cortical PV-positive interneuron

To visualize the Cre recombinase-positive PV interneurons *ex vivo*, the *Grin1* mutant mice (and Ppp1r2cr e mice as controls) were further bred to floxed-tdTomato mice (Ai14, JAX #007914) and Gad1 promotor-EGFP (line G42, JAX #007677) mice. The genotype of mutant quadruple transgenic mouse was Ppp1r2cre(cre/+):Grin1(flox/flox):tdTomato(flox/+):G42-GFP(+/-), and the floxed-control triple transgenic mouse was Ppp1r2cre(cre/+):tdTomato(flox/+):G42-GFP(+/-). Since G42-GFP is expressed exclusively in the PV-positive interneurons (Chattopadhyaya B et al, 2004), double-positive neurons for tdTomato (*i.e.,* Cre-positive)/EGFP in the cortex were treated as *Grin1*-deleted PV interneurons.

PCR genotyping for floxed-Grin1 line

PCR was carried out with GoTaq DNA polymerase with 5xBuffer (Promega Corp); additionally, one PCR volume contained 0.5 mM $MgCl_2$, 0.1 µM dNTPs, 0.5 µM NR1loxP2 primer (ACAATAGAGATTCAAGGCTGATCAAGG), 0.5 µM NR1loxP3 primer (CTCTGGGTGGCTTGCCTGGCTGTATGTT), and 1.0 µl of sample DNA (1 µg/µl). The PCR was performed on a ProFlex PCR Thermal Cycler (Applied Biosciences) with the following protocol: one cycle of 95°C for 2 min; 35 cycles of 95°C for 10 sec; 51°C for 45 sec, 72°C for 1 min, and one cycle of 72°C for 5 min.

Ex vivo whole-cell patch-clamp recording

Brain Slice preparation

Before slice preparation, some animals individually received the 40-Hz ASSR click-train stimuli under the same protocol as applied during *in vivo* LFP recording experiments in a sound-proof chamber and compared with the littermates with no click-train stimuli. Within 10-15 min after the cessation of acoustic stimuli, coronal brain slices containing auditory cortex were prepared from 4-6 week old mice (both sexes) as described in Nakao K et al. (2020). Briefly, after rapid decapitation under isoflurane anesthesia the brain was quickly removed and placed in ice cooled slicing aCSF, containing (in mM) 234 Sucrose, 2.5 KCl, 25 $NaHCO_3$, 1.25 $NaHPO_4$, 25 Glucose, 0.5 $CaCl_2$, 7 $MgCl_2$, 3 Myo-inositol, 2 Na-pyruvate, and 0.4 ascorbic acid; pH 7.4. aCSF was continuously bubbled with carbogen gas (95% O_2 – 5% CO_2). The brain was cut into a block of about 1 cm and mounted on the cutting platform of compresstome vibrating microtome (VF-310-0Z, Precisionary Instruments, USA) for slicing. Slices (350 µm-thickness) were incubated at 35°C in recording aCSF containing (in mM) 125 NaCl, 2.5KCl, 25 $NaHCO_3$, 1.25 $NaHPO_4$, 25 Glucose, 2 $CaCl_2$, 1 $MgCl_2$, 3 Myo-inositol, 2 Na-pyruvate, and 0.4 ascorbic acid, pH 7.4, for 30 min and then kept at room temperature until used for the experiments.

Patch clamp electrophysiology

Whole cell voltage/current clamp recording were conducted from layer(L)2/3 pyramidal and PV interneurons (double positive for tdTomato and GFP in the quadruple transgenic mice). Brain slices were continuously perfused with aCSF at flow rate of 2-3 ml per min and mounted on a recording chamber for electrophysiological recording. Patch pipettes were pulled with Flaming Brown micropipette puller (P97, Sutter Instrument Co. USA) and had resistance of 3-5 MΩ when filled with intracellular buffer. Buffer composition for current clamp recording was, in mM: 140 K-gluconate, 10 HEPES, 4 Mg-ATP, 0.4 Na-GTP, 10 Na-Phosphocreatine pH adjusted to 7.3 with KOH. For the voltage clamp recording K-gluconate was replaced with 140 mM CsCl and 5 mM QX-314 was added. Action potentials were recorded in current clamp mode as a function of injected current from -200 pA to 400

pA with step of 25 pA. To record spontaneous inhibitory postsynaptic current (sIPSC), whole-cell voltage-clamp recordings were done on L2/3 pyramidal neurons holding the membrane potential at -70 mV. Series resistance was in all cases <20 MΩ and not compensated. IPSC were isolated by adding 50µM D-2-Amino-5-phosphopentanoic acid (D-AP5, Alomone labs) and 20 µM 6-Cyano-7-nitroquinoxaline-2,3-dione (CNQX, Alomone labs) to the recording aCSF. All recordings were done at 30-32 ^{o}C using EPC10 USB Quadro (HEKA Instruments Inc. USA). IPSCs were detected and analyzed using custom written Igor procedures by setting event detection threshold at 5 pA. To achieve the targeted pharmacological manipulation, following chemicals were bath-applied for minimum of 10 min during the electrophysiological recording: selective 5-HT2AR antagonist M100907 (2 µM, Sigma); selective 5-HT2AR agonist TCB-2 (20 µM, Tocris); selective 5-HT2CR agonist MK212 (2 µM, Tocris); $G\alpha_q$-selective G protein inhibitor BIM-46187 (50 µM, Calbiochem); selective GIRK1/2 channel activator ML297 (10 µM, Tocris); $GABA_A$ receptor antagonist (+)-Bicuculline (20 µM, Alomone Labs).

Immunohistochemistry

Immunohistochemistry was performed as previously described (Belforte et al, 2010). Briefly, mice (n=3 for each genotype) at 2-3 months of age were anesthetized with Isoflurane and transcardially perfused with 4% paraformaldehyde (PFA) in PBS followed by postfixed in 4% PFA/PBS for one hr. Coronal brain sections (35 µm thickness) containing auditory cortex were double-immunostained with rabbit anti-5-HT2AR (1:200, 24288, Immunostar, USA) and mouse anti-PV (1:5000, 235, Swant, Switzerland) at 4 ^{o}C for 24 hr. The specificity of this antibody to mouse 5-HT2AR was validated using 5--HT2AR Knockout mouse brain tissue (Hamor PU et al, Pharmacol Biochem Behav 175, 89-100, 2018). Anti-PV immunoreactivity was used to assess the degree of 5-HT2AR levels in the Grin1-deleted fast-spiking interneurons, because cre-targeted neurons represent 83.5% of PV-positive interneurons in cortical layer 2/3 (Fig S10, Belforte JE et al, Nat Neurosci 13:76-83, 2010). Anti-5-HT2AR and anti-PV were visualized by staining with Alexa594 goat Anti-Rabbit IgG (1:400, A-11037, Invitrogen) and Alexa488-goat Anti-Mouse IgG (1:400, A11029, Invitrogen), respectively. Images of the auditory cortex were captured using confocal microscopy system (Nikon A1). NIH ImageJ software was used to measure the integrated density of 5-HT2AR in PV-positive interneurons or non-PV neurons. The corrected total cell fluorescence (CTCF) was calculated as previously described (McCloy RA et al, 2014). CTCF = Integrated Density – (Area of selected cell X Mean fluorescence of background readings).

FISH

Fluorescence in situ hybridization (FISH) was performed as previously described (McMeekin LJ et al, 2018) using the RNAscope Multiplex Fluorescent assay (Advanced Cell Diagnostics) according to the manufacturer's instructions. Mice at 3 months of age (n = 3/genotype) were briefly anesthetized with isoflurane and decapitated and brains were removed and frozen with powdered dry ice for sectioning on a cryostat. Then, 20 µm sections were collected on SuperFrost Plus slides (Thermo Fisher Scientific) and immediately refrozen. Samples were fixed in 4% prechilled paraformaldehyde followed by dehydration in ethanol and pretreatment in protease IV (Advanced Cell Diagnostics). Probes were designed by Advanced Cell Diagnostics USA to recognize exons of murine htr2a. Colocalization studies were performed using probe for Pvalb (Advanced Cell Diagnostics). Tissues were treated with a mixture of probes for 2 h at 40°C followed by fluorescent amplification and mounting with Prolong gold antifade mounting medium containing DAPI (Thermo Fisher Scientific). Images were captured with a Nikon A1+ confocal microscope, and mean gray values were measured on the PV-positive neurons by NIH ImageJ software.

Supplementary Figures

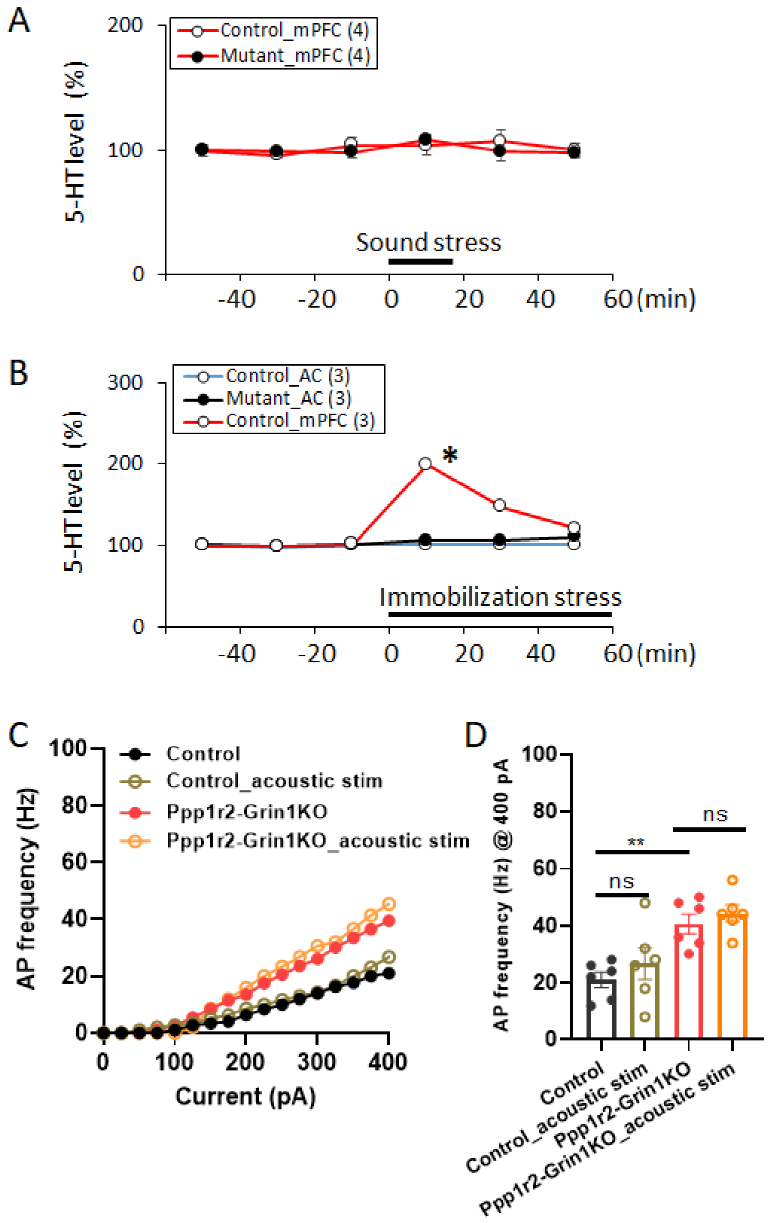

Fig. S1 (**A**) *In vivo* awake microdialysis revealed that both control (n = 4) and mutant (n = 4) mice showed no increase in extracellular 5-HT concentration in medial PFC (mPFC) during 40-Hz auditory steady-state response (ASSR) stimuli (acoustic stimuli). (**B**) In contrast, immobilization stress in plastic bags (Braintree Scientific Inc, USA) for 60 min induced 5-HT increase in mPFC of control mice (n =3), but not in auditory cortex (AC) of control (n = 3) and mutant (n = 3) mice (Control_AC vs Control_mPFC, *p< 0.05. Student's t-test). These results suggested that (1) the data in (A) showing no increase in 5-HT in mPFC by acoustic stimuli is unlikely to be experimental error. And (2) 5-HT release in the cortical area may be regulated at serotonergic axon terminals, depending on the kinds of stress stimuli. (**C** and **D**) Effect of acoustic stimulation on AP firing *ex vivo* on layer 2/3 pyramidal neurons from mPFC slices. Mean AP frequency as function of current injection with and without *in vivo* acoustic stimulation in control and mutant mice. (**D**) Summary plot of AP frequency firing in response to 400 pA-current injection. Acoustic stimuli showed no effect on the AP frequency regardless of genotypes, which is consistent with the data in (A) showing acoustic stimuli fail to increase 5-HT in mPFC. Data presented in D is mean ± sem from indicated number of neurons, in parenthesis, from 4 control and 3 mutant mice (both sexes), **p < 0.001. One-way ANOVA.

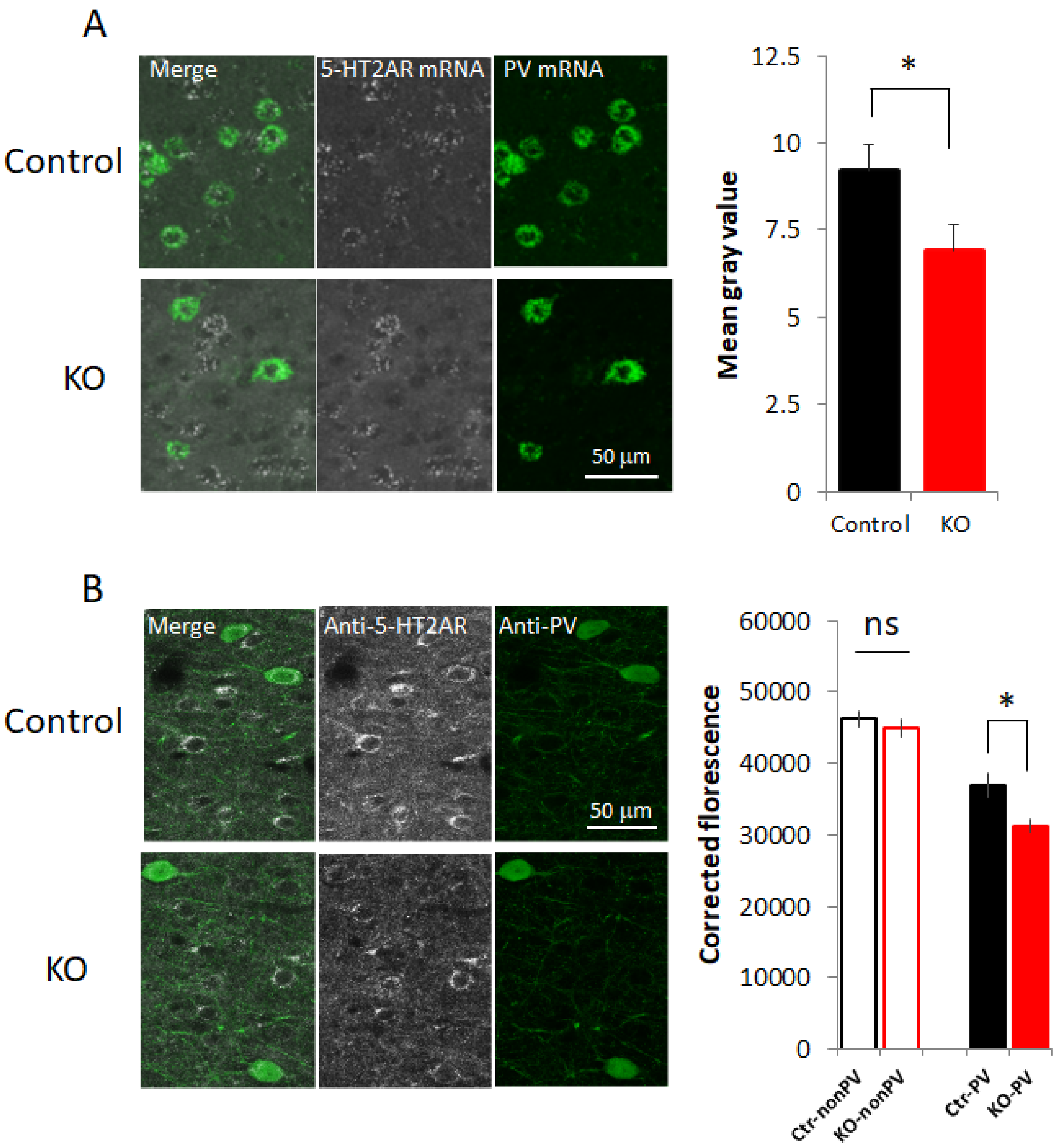

Fig. S2 (**A**) Transcriptional levels of Htr2a gene (white), which encodes 5-HT2AR, in the somata of cortical PV-positive interneurons (green) were reduced in the *Grin1* mutant mice (n=3) compared to the floxed-control mice (n=3). $p^* < 0.04$, Student *t*-test. (**B**) 5-HT2AR-Immunoreactivity levels (white) in the somata of cortical PV-positive interneurons (green) were reduced in the *Grin1* mutants (n=3) relative to floxed-controls (n=3). $p^* < 0.01$, Student *t*-test. Note that there is no difference in 5-HT2AR-IR of non-PV neurons, mostly pyramidal neurons.

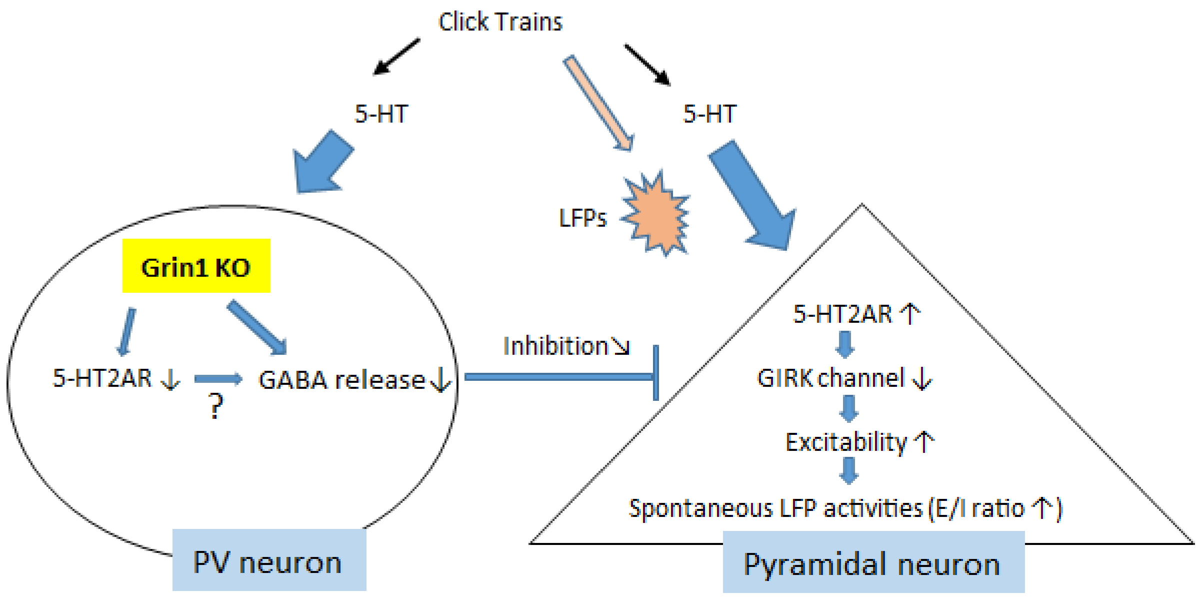

Fig. S3 Schematic model to describe how acoustic stimuli induces 5-HT2AR-mediated hyperexcitability in pyramidal neurons in the auditory cortex following NMDAR hypofunction in PV neurons. Acoustic stimuli (ASSR click trains) releases 5-HT activity-dependently in the auditory cortex. *Grin1* deletion in PV interneurons may undergo deficits in presynaptic GABA release not only in a 5-HT-dependent manner, but also at the baseline level. Such impaired inhibition in early postnatal development dysregulates 5-HT2ARs in pyramidal neurons. Upon acoustic stimuli, activation of dysregulated 5-HT2ARs results in activation of Gαq protein and suppression of GIRK channels, leading to a hyper-excitable state in the auditory cortex. Note that specific contribution of 5-HT2AR down-regulation in PV interneurons to the disinhibition of pyramidal neurons remains to be determined. LFP: local field potential.

Table S1: Membrane properties of layer2/3 pyramidal neurons in Grin1 mutant mice

Parameter	Floxed control	Floxed control_Acoustic stimulation	Grin1-KO	Grin1-KO_ acoustic stimulation
**RMP, mV**	77.88 ± 2.03	77.75 ± 2.03	78 ± 2.03	84.29 ± 2.03
**Time constant, ms**	13.05 ± 2.35	12.13 ± 1.18	16.35 ± 2.66	13.86 ± 2.00
**Membrane Capacitance, pF**	71.7 ± 14.17	64.86 ± 7.44	94.69 ± 13.72	97.12 ± 13.97
**Input Resistance, MΩ**	196.4 ± 30.23	192 ± 17.29	186.3 ± 27.33	157.1 ± 16.76
**AP threshold, mV**	45.13 ± 1.31	45 ± 1.94	47.13 ± 1.62	41.86 ± 1.52
**AP Amplitude, mV**	91.56 ± 4.2	88.25 ± 4.38	91.5 ± 4.19	96.57 ± 3.3
**AHP, mV**	10.71 ± 1.12	11.78 ± 1.07	14.4 ± 1.44	11.43 ± 1.37

Note that there was no alteration in all the parameters examined between the genotypes and before and after *in vivo* acoustic stimulation. RMP, resting membrane potential; AP, action potential; AHP, after-hyperpolarization.

Supplementary References

Belforte, J.E., et al., *Postnatal NMDA receptor ablation in corticolimbic interneurons confers schizophrenia-like phenotypes.* Nat Neurosci, 2010;**13**(1):76-83.

Chattopadhyaya B, Di Cristo G, Higashiyama H, Knott GW, Kuhlman SJ, Welker E, et al*. Experience and activity-dependent maturation of perisomatic GABAergic innervation in primary visual cortex during a postnatal critical period.* J Neurosci. 2004;**24**(43):9598-611.

Hámor PU, Šírová J, Páleníček T, Zaniewska M, Bubeníková-Valešová V, Schwendt M. Chronic methamphetamine self-administration dysregulates 5-HT2A and mGlu2 receptor expression in the rat prefrontal and perirhinal cortex: Comparison to chronic phencyclidine and MK-801.2018;**175**:89-100.

McCloy RA, Rogers S, Caldon CE, Lorca T, Castro A, Burgess A. *Partial inhibition of Cdk1 in G2 phase overrides the SAC and decouples mitotic events.* Cell Cycle. 2014;**13**:1400–1412.

McMeekin LJ, Li Y, Fox SN, Rowe GC, Crossman DK, Day JJ, Li Y, Detloff PJ, Cowell RM. *Cell-Specific Deletion of PGC-1α from Medium Spiny Neurons Causes Transcriptional Alterations and Age-Related Motor Impairment.* J Neurosci. 2018; **38**(13):3273-3286.

Nakao K, Singh M, Sapkota K, Hagler BC, Hunter RN, Raman C, et al. *GSK3β inhibition restores cortical gamma oscillation and cognitive behavior in a mouse model of NMDA receptor hypofunction relevant to schizophrenia*. Neuropsychopharmacology. 2020; **45**(13):2207-2218.
